# 2-[1-(3-Amino­phenyl­imino)­eth­yl]phenol

**DOI:** 10.1107/S1600536811017624

**Published:** 2011-05-14

**Authors:** Anita Blagus, Branko Kaitner

**Affiliations:** a Department of Chemistry, J. J. Strossmayer University, Osijek, Franje Kuhača 20, HR-31000 Osijek, Croatia; b Laboratory of General and Inorganic Chemistry, Department of Chemistry, Faculty of Science, University of Zagreb, Horvatovac 102a, HR-10002 Zagreb, Croatia

## Abstract

The title compound, C_14_H_14_N_2_O, exists as the enol–imine tautomer. A strong intra­molecular hydrogen bond between O and N atoms forms a six-membered ring with an *S*(6) graph-set motif, which is approximately coplanar with the phenol ring, the inter­planar angle being 3.4 (3)°. In the crystal, inter­molecular C—H⋯O hydrogen bonds and N—H⋯π inter­actions link the mol­ecules into infinite chains along [100].

## Related literature

For background to Schiff base compounds, see: Blagus & Kaitner (2007 ▶); Blagus *et al.* (2010 ▶). For the photochromic and thermochromic characteristics of Schiff bases, see: Hadjoudis & Mavridis (2004 ▶). For graph-set notation of hydrogen bonds, see Bernstein *et al.* (1995 ▶). For standard bond lengths, see: Allen *et al.* (1987 ▶).
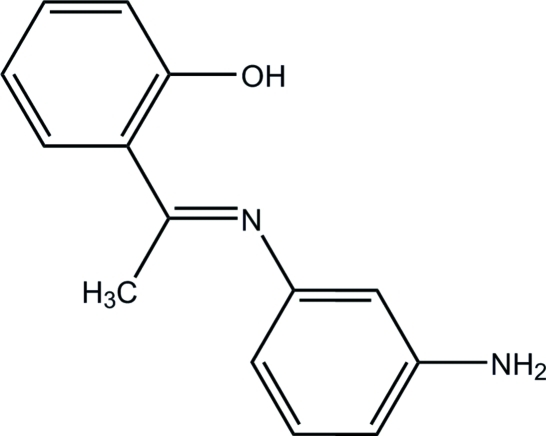


## Experimental

### 

#### Crystal data

C_14_H_14_N_2_O
*M* *_r_* = 226.27
Orthorhombic, 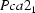

*a* = 9.0625 (2) Å
*b* = 5.5777 (2) Å
*c* = 23.2349 (6) Å
*V* = 1174.48 (6) Å^3^
*Z* = 4
Mo *K*α radiation
μ = 0.08 mm^−1^
*T* = 298 K
0.6 × 0.5 × 0.4 mm

#### Data collection

Oxford Diffraction Xcalibur CCD diffractometer
6829 measured reflections
1317 independent reflections
1145 reflections with *I* > 2σ(*I*)
*R*_int_ = 0.016

#### Refinement

*R*[*F* ^2^ > 2σ(*F* ^2^)] = 0.030
*wR*(*F* ^2^) = 0.087
*S* = 1.10
1317 reflections
165 parameters
1 restraint
H atoms treated by a mixture of independent and constrained refinement
Δρ_max_ = 0.15 e Å^−3^
Δρ_min_ = −0.11 e Å^−3^

### 

Data collection: *CrysAlis CCD* (Oxford Diffraction, 2003 ▶); cell refinement: *CrysAlis RED* (Oxford Diffraction, 2003 ▶); data reduction: *CrysAlis RED*; program(s) used to solve structure: *SHELXS97* (Sheldrick, 2008 ▶); program(s) used to refine structure: *SHELXL97* (Sheldrick, 2008 ▶); molecular graphics: *ORTEP-3* (Farrugia, 1997 ▶); software used to prepare material for publication: *WinGX* (Farrugia, 1999 ▶), *PARST97* (Nardelli, 1995 ▶) and *Mercury* (Macrae *et al.*, 2006 ▶).

## Supplementary Material

Crystal structure: contains datablocks I, global. DOI: 10.1107/S1600536811017624/fy2009sup1.cif

Structure factors: contains datablocks I. DOI: 10.1107/S1600536811017624/fy2009Isup3.hkl

Supplementary material file. DOI: 10.1107/S1600536811017624/fy2009Isup3.cml

Additional supplementary materials:  crystallographic information; 3D view; checkCIF report

## Figures and Tables

**Table 1:** Hydrogen-bond geometry (Å, °) *Cg* is the centroid of the C9–C14 ring.

*D*—H⋯*A*	*D*—H	H⋯*A*	*D*⋯*A*	*D*—H⋯*A*
O1—H1⋯N1	1.04 (4)	1.59 (4)	2.540 (2)	150 (3)
C8—H8*B*⋯O1^i^	0.96	2.71	3.243 (3)	116
N2—H1*N*2⋯*Cg*^i^	0.90 (4)	2.71 (4)	3.457 (3)	142 (3)

Symmetry code: (i) 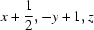.

**Fig. 1.**
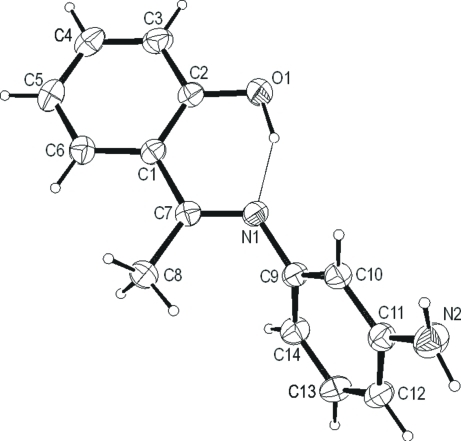
ORTEPIII molecular structure of (I) showing our atom-labelling scheme. Thermal ellipsoids are drawn at the 50% probability level. The intramolecular hydrogen bonds O—H···N is shown as thin line.

**Fig. 2.**
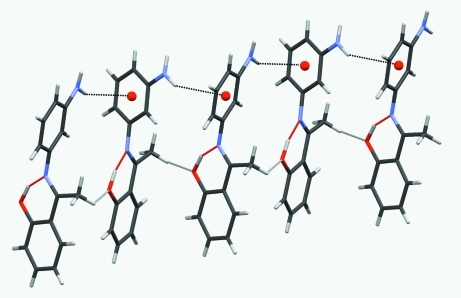
Chains formed in the crystals of the title compound along the [100] direction. All intermolecular interactions are represented by dotted lines. The red spheres represent the centroids of C9—C14 aromatic rings.

## supplementary crystallographic information

### Comment

Schiff bases are some of the most widely used chelating ligands in the field of metal-organic coordination chemistry (Blagus *et al.*, 2010). The Schiff bases derived from *ortho* hydroxy aldehydes or ketons and aromatic diamines often have photochromic and thermochromic characteristics (Hadjoudis & Mavridis 2004). In this work we report the preparation and the crystal and molecular structure of a novel ketimine Schiff base 2-[1-(3-aminophenylimino)ethyl]phenol (Scheme 1).

The presence of intramolecular O1–H···N1 hydrogen bond [2.540 (2) Å] shows unequivocally that the molecular conformation of compound (1) in the crystalline state is in the enol-imino form. As shown in Figure 2, the Schiff base molecules link mutually in an one-dimensional chain forming a graph-set motif *C*(5) in the notation of Bernstein *et al.*, (1995) along the [100] direction through a C–H···O [3.243 (3) Å] intermolecular hydrogen bond. Another intermolecular connection between the same neighbouring molecules forms through the terminal primary N2-amino group N–H···π interaction [3.457 (3) Å; π refers to the C9—C14 aromatic system centroid). All bond lengths are within the standard values (Allen *et al.*, 1987) and are comparable with the similar ketimine Schiff bases as cited above (Blagus & Kaitner, 2007).

### Experimental

The title compound was prepared by refluxing a methanolic solution of *m*-phenylendiamine (540 mg, 5 mmol) and 2-hydroxyacetophenone (1.25 ml, 10 mmol) for 4 h at the temperature of 80 °C. The water formed during the reaction was removed by a Dean-Stark trap. After cooling, the brown solid precipitate was filtered. Diffraction quality crystals were obtained by slow evaporation from ether solution.

### Refinement

All N- and O-bound H atoms were located in the difference Fourier map. The position and the isotropic thermal parameters of N-bound H atoms were refined, while the O-bound H atom was treated as riding atom. Aromatic H atoms were placed in calculated positions and treated as riding on their parent C atoms with C—H = 0.93 Å and *U*_iso_(H) = 1.2 *U*_eq_(C) for Csp2. In the absence of significant anomalous scattering effects Friedel pairs have been merged.

### Crystal data

**Table:**

C_14_H_14_N_2_O	*F*(000) = 480
*M**_r_* = 226.27	*D*_x_ = 1.280 Mg m^−^^3^
Orthorhombic, *P**c**a*2_1_	Mo *K*α radiation, λ = 0.71069 Å
Hall symbol: P 2c -2ac	Cell parameters from 1145 reflections
*a* = 9.0625 (2) Å	θ = 4–27°
*b* = 5.5777 (2) Å	µ = 0.08 mm^−^^1^
*c* = 23.2349 (6) Å	*T* = 298 K
*V* = 1174.48 (6) Å^3^	Prism, yellow
*Z* = 4	0.6 × 0.5 × 0.4 mm

### Data collection

**Table:**

Oxford Diffraction Xcalibur CCD diffractometer	1145 reflections with *I* > 2σ(*I*)
Radiation source: fine-focus sealed tube	*R*_int_ = 0.016
graphite	θ_max_ = 27.0°, θ_min_ = 4.1°
ω scans	*h* = −11→11
6829 measured reflections	*k* = −7→5
1317 independent reflections	*l* = −29→28

### Refinement

**Table:**

Refinement on *F*^2^	Secondary atom site location: difference Fourier map
Least-squares matrix: full	Hydrogen site location: inferred from neighbouring sites
*R*[*F*^2^ > 2σ(*F*^2^)] = 0.030	H atoms treated by a mixture of independent and constrained refinement
*wR*(*F*^2^) = 0.087	*w* = 1/[σ^2^(*F*_o_^2^) + (0.0469*P*)^2^ + 0.130*P*] where *P* = (*F*_o_^2^ + 2*F*_c_^2^)/3
*S* = 1.10	(Δ/σ)_max_ < 0.001
1317 reflections	Δρ_max_ = 0.15 e Å^−^^3^
165 parameters	Δρ_min_ = −0.11 e Å^−^^3^
1 restraint	Extinction correction: *SHELXL97* (Sheldrick, 2008), Fc^*^=kFc[1+0.001xFc^2^λ^3^/sin(2θ)]^-1/4^
Primary atom site location: structure-invariant direct methods	Extinction coefficient: 0.027 (5)

### Special details

**Table:**

Geometry. All e.s.d.'s (except the e.s.d. in the dihedral angle between two l.s. planes) are estimated using the full covariance matrix. The cell e.s.d.'s are taken into account individually in the estimation of e.s.d.'s in distances, angles and torsion angles; correlations between e.s.d.'s in cell parameters are only used when they are defined by crystal symmetry. An approximate (isotropic) treatment of cell e.s.d.'s is used for estimating e.s.d.'s involving l.s. planes.
Refinement. Refinement of *F*^2^ against ALL reflections. The weighted *R*-factor *wR* and goodness of fit *S* are based on *F*^2^, conventional *R*-factors *R* are based on *F*, with *F* set to zero for negative *F*^2^. The threshold expression of *F*^2^ > σ(*F*^2^) is used only for calculating *R*-factors(gt) *etc*. and is not relevant to the choice of reflections for refinement. *R*-factors based on *F*^2^ are statistically about twice as large as those based on *F*, and *R*- factors based on ALL data will be even larger.

### Fractional atomic coordinates and isotropic or equivalent isotropic displacement parameters (Å^2^)

**Table:**

	*x*	*y*	*z*	*U*_iso_*/*U*_eq_	
O1	0.41349 (19)	0.5690 (3)	0.29685 (8)	0.0596 (5)	
H1	0.468 (4)	0.505 (6)	0.2607 (17)	0.089*	
N1	0.5638 (2)	0.3031 (3)	0.23025 (7)	0.0440 (4)	
N2	0.8305 (3)	0.5261 (4)	0.05762 (11)	0.0656 (6)	
C1	0.5491 (2)	0.2236 (4)	0.33034 (9)	0.0402 (4)	
C2	0.4534 (2)	0.4203 (4)	0.33965 (9)	0.0448 (5)	
C3	0.3969 (3)	0.4621 (5)	0.39453 (11)	0.0588 (6)	
H3	0.3360	0.5937	0.4008	0.071*	
C4	0.4300 (3)	0.3117 (5)	0.43923 (11)	0.0638 (7)	
H4	0.3891	0.3393	0.4753	0.077*	
C5	0.5235 (3)	0.1192 (5)	0.43119 (10)	0.0619 (6)	
H5	0.5473	0.0195	0.4619	0.074*	
C6	0.5814 (3)	0.0761 (4)	0.37718 (10)	0.0515 (5)	
H6	0.6435	−0.0548	0.3719	0.062*	
C7	0.60706 (19)	0.1706 (4)	0.27231 (9)	0.0395 (4)	
C8	0.7137 (2)	−0.0334 (4)	0.26549 (11)	0.0510 (5)	
H8A	0.6640	−0.1821	0.2730	0.076*	
H8B	0.7937	−0.0141	0.2922	0.076*	
H8C	0.7516	−0.0344	0.2269	0.076*	
C9	0.6061 (2)	0.2535 (4)	0.17224 (9)	0.0426 (4)	
C10	0.6998 (2)	0.4088 (4)	0.14395 (9)	0.0452 (5)	
H10	0.7375	0.5414	0.1633	0.054*	
C11	0.7386 (2)	0.3685 (4)	0.08652 (9)	0.0458 (4)	
C12	0.6800 (2)	0.1690 (4)	0.05828 (10)	0.0509 (5)	
H12	0.7045	0.1396	0.0201	0.061*	
C13	0.5863 (3)	0.0158 (5)	0.08685 (10)	0.0548 (5)	
H13	0.5486	−0.1172	0.0677	0.066*	
C14	0.5470 (3)	0.0563 (4)	0.14380 (11)	0.0514 (5)	
H14	0.4823	−0.0468	0.1626	0.062*	
H1N2	0.874 (4)	0.635 (7)	0.0803 (14)	0.077*	
H2N2	0.864 (4)	0.481 (6)	0.0229 (18)	0.077*	

### Atomic displacement parameters (Å^2^)

**Table:**

	*U*^11^	*U*^22^	*U*^33^	*U*^12^	*U*^13^	*U*^23^
O1	0.0719 (11)	0.0573 (10)	0.0494 (9)	0.0216 (8)	0.0010 (8)	0.0010 (8)
N1	0.0465 (9)	0.0483 (9)	0.0371 (8)	0.0038 (8)	−0.0003 (7)	−0.0028 (7)
N2	0.0719 (14)	0.0692 (14)	0.0558 (12)	−0.0132 (12)	0.0135 (11)	0.0016 (11)
C1	0.0351 (8)	0.0468 (10)	0.0388 (9)	−0.0034 (8)	−0.0023 (8)	−0.0033 (9)
C2	0.0452 (10)	0.0489 (11)	0.0403 (10)	0.0001 (9)	−0.0012 (9)	−0.0043 (9)
C3	0.0604 (13)	0.0658 (15)	0.0501 (13)	0.0077 (12)	0.0057 (11)	−0.0125 (11)
C4	0.0667 (15)	0.0847 (19)	0.0399 (11)	−0.0016 (14)	0.0037 (11)	−0.0071 (12)
C5	0.0690 (15)	0.0746 (16)	0.0422 (12)	−0.0035 (13)	−0.0036 (11)	0.0072 (12)
C6	0.0516 (12)	0.0554 (12)	0.0476 (12)	0.0028 (10)	−0.0052 (10)	0.0019 (10)
C7	0.0344 (8)	0.0432 (10)	0.0408 (10)	−0.0028 (8)	−0.0019 (8)	−0.0030 (8)
C8	0.0471 (11)	0.0523 (11)	0.0535 (12)	0.0087 (9)	0.0006 (10)	−0.0015 (10)
C9	0.0435 (9)	0.0474 (10)	0.0369 (9)	0.0052 (9)	−0.0014 (8)	−0.0037 (8)
C10	0.0480 (10)	0.0438 (10)	0.0439 (10)	−0.0002 (9)	−0.0042 (9)	−0.0038 (9)
C11	0.0429 (9)	0.0512 (10)	0.0435 (10)	0.0038 (9)	−0.0001 (8)	0.0014 (9)
C12	0.0527 (12)	0.0614 (13)	0.0385 (10)	0.0034 (11)	0.0016 (9)	−0.0074 (10)
C13	0.0600 (13)	0.0576 (12)	0.0467 (12)	−0.0058 (11)	−0.0009 (10)	−0.0142 (11)
C14	0.0517 (12)	0.0537 (12)	0.0489 (11)	−0.0068 (10)	0.0020 (10)	−0.0039 (10)

### Geometric parameters (Å, °)

**Table:**

O1—C2	1.345 (3)	C5—H5	0.9300
O1—H1	1.04 (4)	C6—H6	0.9300
N1—C7	1.286 (3)	C7—C8	1.501 (3)
N1—C9	1.428 (3)	C8—H8A	0.9600
N2—C11	1.385 (3)	C8—H8B	0.9600
N2—H1N2	0.89 (4)	C8—H8C	0.9600
N2—H2N2	0.90 (4)	C9—C10	1.380 (3)
C1—C6	1.395 (3)	C9—C14	1.391 (3)
C1—C2	1.415 (3)	C10—C11	1.398 (3)
C1—C7	1.477 (3)	C10—H10	0.9300
C2—C3	1.394 (3)	C11—C12	1.397 (3)
C3—C4	1.368 (4)	C12—C13	1.376 (3)
C3—H3	0.9300	C12—H12	0.9300
C4—C5	1.380 (4)	C13—C14	1.389 (3)
C4—H4	0.9300	C13—H13	0.9300
C5—C6	1.381 (4)	C14—H14	0.9300
			
C2—O1—H1	105 (2)	C1—C7—C8	118.49 (18)
C7—N1—C9	121.61 (17)	C7—C8—H8A	109.5
C11—N2—H1N2	114 (2)	C7—C8—H8B	109.5
C11—N2—H2N2	117 (2)	H8A—C8—H8B	109.5
H1N2—N2—H2N2	125 (3)	C7—C8—H8C	109.5
C6—C1—C2	117.80 (19)	H8A—C8—H8C	109.5
C6—C1—C7	121.29 (18)	H8B—C8—H8C	109.5
C2—C1—C7	120.85 (18)	C10—C9—C14	120.5 (2)
O1—C2—C3	118.3 (2)	C10—C9—N1	119.53 (18)
O1—C2—C1	122.01 (19)	C14—C9—N1	119.9 (2)
C3—C2—C1	119.7 (2)	C9—C10—C11	120.56 (19)
C4—C3—C2	120.7 (2)	C9—C10—H10	119.7
C4—C3—H3	119.6	C11—C10—H10	119.7
C2—C3—H3	119.6	N2—C11—C12	120.4 (2)
C3—C4—C5	120.6 (2)	N2—C11—C10	120.8 (2)
C3—C4—H4	119.7	C12—C11—C10	118.8 (2)
C5—C4—H4	119.7	C13—C12—C11	120.2 (2)
C4—C5—C6	119.5 (2)	C13—C12—H12	119.9
C4—C5—H5	120.3	C11—C12—H12	119.9
C6—C5—H5	120.3	C12—C13—C14	121.1 (2)
C5—C6—C1	121.7 (2)	C12—C13—H13	119.4
C5—C6—H6	119.1	C14—C13—H13	119.4
C1—C6—H6	119.1	C13—C14—C9	118.9 (2)
N1—C7—C1	118.05 (17)	C13—C14—H14	120.6
N1—C7—C8	123.46 (19)	C9—C14—H14	120.6
			
C6—C1—C2—O1	−178.5 (2)	C6—C1—C7—C8	−5.7 (3)
C7—C1—C2—O1	−1.2 (3)	C2—C1—C7—C8	177.18 (17)
C6—C1—C2—C3	0.9 (3)	C7—N1—C9—C10	−111.9 (2)
C7—C1—C2—C3	178.1 (2)	C7—N1—C9—C14	71.2 (3)
O1—C2—C3—C4	177.8 (2)	C14—C9—C10—C11	−0.9 (3)
C1—C2—C3—C4	−1.6 (4)	N1—C9—C10—C11	−177.75 (19)
C2—C3—C4—C5	1.9 (4)	C9—C10—C11—N2	178.9 (2)
C3—C4—C5—C6	−1.4 (4)	C9—C10—C11—C12	0.3 (3)
C4—C5—C6—C1	0.7 (4)	N2—C11—C12—C13	−178.6 (2)
C2—C1—C6—C5	−0.4 (3)	C10—C11—C12—C13	−0.1 (3)
C7—C1—C6—C5	−177.7 (2)	C11—C12—C13—C14	0.5 (4)
C9—N1—C7—C1	−175.40 (18)	C12—C13—C14—C9	−1.0 (4)
C9—N1—C7—C8	5.0 (3)	C10—C9—C14—C13	1.3 (3)
C6—C1—C7—N1	174.69 (19)	N1—C9—C14—C13	178.1 (2)
C2—C1—C7—N1	−2.5 (3)		

### Hydrogen-bond geometry (Å, °)

**Table:**

Cg is the centroid of the C9–C14 ring.

**Table:**

*D*—H···*A*	*D*—H	H···*A*	*D*···*A*	*D*—H···*A*
O1—H1···N1	1.04 (4)	1.59 (4)	2.540 (2)	150 (3)
C8—H8B···O1^i^	0.96	2.71	3.243 (3)	116
N2—H1N2···Cg^i^	0.90 (4)	2.71 (4)	3.457 (3)	142 (3)

Symmetry codes: (i) *x*+1/2, −*y*+1, *z*.
